# Neonatal tetanus in St. Mary’s Hospital Lacor: A case report

**DOI:** 10.1002/ccr3.3091

**Published:** 2020-07-18

**Authors:** Ronald Okidi, Vanusa Da Consolação Sambo, Jacob Eyul

**Affiliations:** ^1^ Department of Surgery St. Mary’s Hospital Lacor Gulu Uganda; ^2^ Department of Paediatrics and Child Health St. Mary’s Hospital Lacor Gulu Uganda; ^3^ Department of Internal medicine St. Mary’s Hospital Lacor Gulu Uganda

**Keywords:** neonatal tetanus, resource‐limited settings, tetanus immunoglobulin, tetanus toxoid

## Abstract

Umbilical stump sepsis in a nonpassively immunized neonate poses a high risk to neonatal tetanus. Management of neonatal tetanus is still a great challenge in resource‐limited settings where some health facilities lack tetanus toxoid containing vaccines, ventilator support, and inaccessibility of tetanus immunoglobulin.

## Graphical abstract

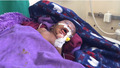

## 1 INTRODUCTION

Tetanus is a life‐threatening disease caused by the anaerobic spore‐forming bacterium, *Clostridium tetani*, which produces a potent neurotoxin responsible for symptoms upon gaining entry through a skin breach in a susceptible host, multiplying under suitable anaerobic environment, hence releasing tetanus toxin (tetanospasmin).^1^, ^2^ Tetanus presents in four main forms: (a) local tetanus, (b) cephalic tetanus and (c) generalized tetanus in adults, and (d) neonatal tetanus.^3^ Neonatal tetanus is almost always fatal.^4^, ^5^

Predisposition to neonatal tetanus includes umbilical stump sepsis, use of unsterile equipment to cut the cord, home delivery, application of cow dung, and other unsafe traditional practices.^6^, ^7^ Failure to suckle breast milk and excessive cry are the early and yet nonspecific manifestation of neonatal tetanus. However, trismus, risus sardonicus, and opisthotonus are characteristic features of generalized neonatal tetanus.^5^

Neonatal tetanus can be prevented possibly by administering vaccines to pregnant or non‐pregnant women, or both, with tetanus toxoid, and through safe clean delivery services.^8^

The World Health Organization (WHO)‐recommended schedules for tetanus toxoid‐containing vaccines (TTCV) include six doses: a three‐dose primary infant series and three booster doses given at ages 12‐23 months, 4‐7 years, and 9‐15 years.^5^ Pregnant women and their newborn babies are protected from tetanus if the mother received six TTCV doses during childhood, or five doses if a catch‐up vaccination schedule was initiated after 1 year of age.^9^ However, newborn infants may develop the disease when their mothers do not have sufficient circulating antibodies to passively protect them.^8^ Mortality from neonatal tetanus is high when symptoms start in those less than 7 days old^10^, ^11^ and are related to autonomic nervous system dysfunction (labile hypertension and unstable heart rate) and spasm of respiratory muscles leading to respiratory failure.^5^ Treatment of neonatal tetanus requires administration of intramuscular 0.5 mL of TTCV and 500 IU of tetanus immunoglobulin on separate thighs and crystalline Penicillin is given for 10 days. An intravenous infusion of diazepam at a dose of 5 mg/kg/d is increased depending on response.^11^

Herein, we present a fatal case of neonatal tetanus in a child born to a first‐time mother who did not receive a single dose of TTCV vaccine during antenatal care (ANC).

## 2 CASE DESCRIPTION

### 2.1 Clinical history

**Figure 1:**
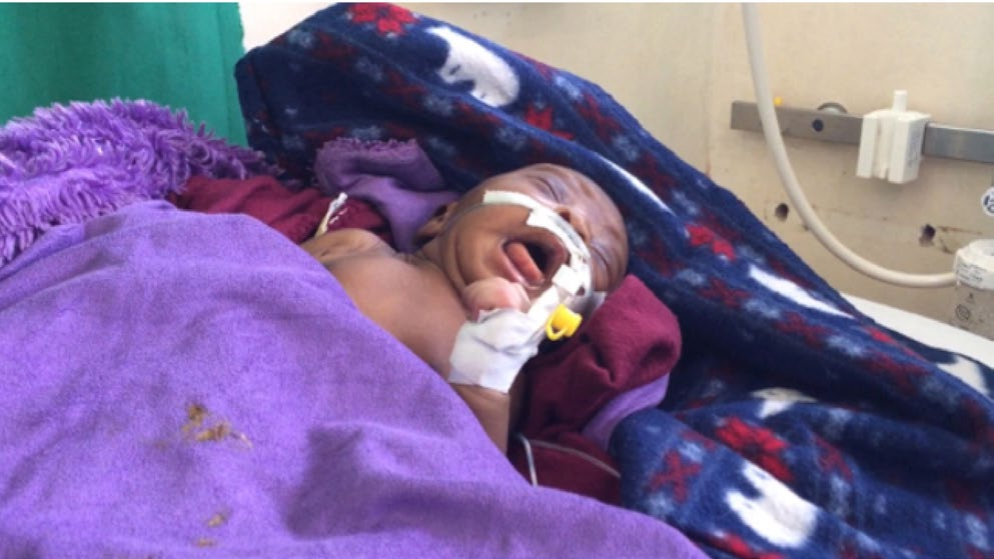
Showing the neonate having an episode of spasm.

A 5‐day‐old male neonate was delivered by spontaneous vertex delivery to a peasant mother in a lower unit health facility (health center III). The baby cried immediately upon delivery and weighed 3.0 kg. He presented with a 3‐day history of high‐grade fever, refusal to breastfeed, excessive crying, and difficulty in breathing. He had episodic provoked spasms (Figure 1). His mother, an 18‐year‐old, para 1 + 0 attended ANC three (3) times and was treated for malaria at 24 weeks of gestation. However, she did not receive the routine TTCV vaccines during all her ANC visits.

### 2.2 Clinical examinations

The baby was sick, on supplemental oxygen via nasal prongs and saturating at 98% on 1 L of oxygen/min. His axillary temperature was 38.2°C, in respiratory distress, respiring at a rate of 72 breaths per min, with bilateral equal breath sounds. Pulse rate was at 121 bpm with normal heart sounds S1 and S2. The umbilical cord stump had a mild purulent discharge. The abdomen was of normal fullness, with a tense abdominal muscle. The rectum had normal stool.

### 2.3 Laboratory investigations

A complete blood count revealed granulocyte and hemoglobin count of 2100 cells/μL of blood (1200‐8000) and 11.5 g/dL (11.5‐16.5), respectively. There was no bacterial growth on blood culture.

### 2.4 Management

The neonate was admitted to the intensive care unit, and initiated on intravenous phenobarbital 10 mg/kg daily, intravenous diazepam 2.5 mg 6‐hourly, intravenous metronidazole 15 mg/kg loading dose followed 7.5 mg/kg bodyweight 12‐hourly and oxygen therapy. The umbilical stump was cleaned using hydrogen peroxide daily and dressed. However, he had no administration of TTCV and tetanus immunoglobulin due to nonavailability and eventually expired on day 7 of life.

## 3 DISCUSSIONS

The WHO aimed at global elimination of neonatal tetanus. The case‐fatality rate of neonatal tetanus without treatment approaches 100%, though with intensive care, this can be decreased to 10%‐20%. The WHO estimated that there were 34 000 neonatal tetanus deaths worldwide in 2015, and St. Mary's hospital Lacor recorded mortality at 78% in 2014.^12^ This neonate presented with typical features of neonatal tetanus^3^ and his umbilical stump was septic, discharging pus, which was most likely the entry route of the deadly *C tetani*.^6^, ^7^ The symptom onset was between the first 24‐48 hours of birth. A short incubation period is associated with a poorer prognosis in both neonatal and adult tetanus cases.^10^, ^11^ Immediate treatment for tetanus upon diagnosis was instituted following the St. Mary's Hospital Lacor Neonatal Tetanus treatment protocol which, included patient stabilization, entry site debridement, rectal diazepam 1.25 mg 4 hourly and whenever necessary with increasing frequency of spasms, intravenous metronidazole 15 mg 6 hourly, tetanus immunoglobulin, oxygen, and nasogastric feeding. However, he did not receive tetanus immunoglobulin, which is known to unbound tetanospasmin, due to its unavailability. This protocol varied slightly from a protocol used in an Indian hospital by not including TTCV and crystalline penicillin to its regimen.^11^, ^13^ On the 3rd day of stay in the ICU, the neonate's condition worsened with severe respiratory distress from respiratory muscle spasms, which is an indicator of autonomic system failure. Phenobarbital was administered with the intent of halting breakthrough spasms but complete suppression of spasms was not attained.^5^

Pregnant mothers routinely receive TTCV during antenatal care visits to prevent neonatal tetanus. Unfortunately, this mother did not receive any dose of TTCV in all her four ANC visits because of the unavailability of the vaccine in the facility where she had been attending ANC from, thus the newborn baby was not protected from this deadly disease.^8^

## 4 CONCLUSION

Umbilical stump sepsis in nonpassively immunized neonates poses a great risk to neonatal tetanus. Management of neonatal tetanus still poses a great challenge in resource‐limited settings where some health facilities lack tetanus toxoid containing vaccines, management that may require ventilator support and inaccessibility of tetanus immunoglobulin.

## CONFLICT OF INTERESTS

All authors declare no conflict of interest.

## AUTHOR CONTRIBUTIONS

RO: actively participated in the management of this neonate in the intensive care unit and significantly contributed in writing the background, discussion of the case, and preparation of the manuscript. VDCS: contributed significantly in the writing the background, discussion, and reviewing the manuscript. JE: contributed significantly in the writing the background, case presentation, and discussion of the case.

## ETHICAL APPROVAL

Lacor Hospital Institutional research and ethics committee approved this case report. The mother of the baby gave a written informed consent and his identity concealed.

## DATA AVAILABLITY STATEMENT

The data of the neonate can be found in St. Mary's Hospital Lacor archive.
